# Nanofibrous Membranes Based on Collagen and Conductive Polymers with Perspective for Biological Applications

**DOI:** 10.3390/membranes15060177

**Published:** 2025-06-11

**Authors:** Tonantzi Pérez-Moreno, Claudia D’Urso, Gabriel Trejo, Maria V. Contreras-Martínez, Omar Lozano, Gerardo J. García-Rivas, Luis G. Arriaga, Gabriel Luna-Barcenas, Janet Ledesma-García

**Affiliations:** 1 Facultad de Ingenieía, División de Investigación y Posgrado, Universidad Autónoma de Querérato, Santiago de Querétaro 76010, Querétaro, Mexico; tonantzi.perez@uaq.mx; 2 Consiglio Nazionale delle Ricerche Istituto di Tecnologie Avanzate per l’Energia “Nicola Giordano”, Via S. Lucia Sopra Contesse, 5, 98126 Messina, Italy; claudia.durso@cnr.it; 3 Centro de Investigación y Desarrollo Tecnológico en Electroquímica, Parque Tecnológico Querétaro-Sanfandila, Pedro Escobedo 76703, Querétaro, Mexico; gtrejo@cideteq.mx (G.T.); mcontreras@cideteq.mx (M.V.C.-M.); 4 Institute for Obesity Research, Escuela de Medicina y Ciencias de la Salud, Cátedra de Cardiología y Medicina Vascular, San Pedro Garza García 66260, Nuevo Leon, Mexico; omar.lozano@tec.mx (O.L.); gdejesus@tec.mx (G.J.G.-R.); 5 The Institute of Advanced Materials for Sustainable Manufacturing, Tecnológico de Monterrey, Santiago de Querétaro 76130, Querétaro, Mexico; gabriel.luna@tec.mx

**Keywords:** collagen fibers, polyaniline, polypyrrole, electrospinning technique

## Abstract

In this study, membranes of collagen–chitosan (C-Ch) in combination with conductive polymers (CPs) such as polyaniline (Pani) and polypyrrole (Ppy) were obtained by electrospinning using non-toxic solvents such as PBS and ethanol. The change in the morphology after swelling was observed by SEM, while an FTIR analysis showed specific interactions between C-Ch and CP. Mechanical tests showed that C-Ch/Ppy exhibited more elastic behavior and a better stress distribution compared to C-Ch/Pani. The diffusion of Na^+^ and Ca^2+^ ions through the membranes was evaluated and showed a greater resistance for Ca^2+^ in both membrane types. Preliminary biocompatibility testing with H9C2 cells showed a successful cell adhesion to the membranes. These results emphasize the potential of C-Ch/Pani composites for electrically active scaffolds and of C-Ch/PPy composites for applications in mechanically dynamic tissue-specific regeneration.

## 1. Introduction

Bioactive membranes, which are able to transport ions and electrical signals, have aroused great interest in recent years due to their potential to restore the functionality of damaged tissue, such as nerves or the heart. In the specific case of the heart, these membranes must have properties that are very similar to those of the native heart muscle, particularly in terms of electrical conductivity. For example, the conductivity of myocardial tissue is measured at 1.6 × 10^−3^ S/cm in the longitudinal direction and 5 × 10^−5^ S/cm in the transverse direction, illustrating the anisotropic nature of the conductivity of the human heart muscle (myocardium) [1]. In addition, the membrane should be able to transport important ions such as Na^+^, K^+^ and Ca^2+^, which are essential for the contraction of the sarcomeres and the proper functioning of the connecting proteins between the cardiac muscle cells [2,3].

Another important property is the modulus of elasticity of the membrane, which must be within the physiological range of the target tissue. In healthy human myocardium, the modulus of elasticity is between 0.2 and 0.5 MPa [4]. This ensures that the scaffold can withstand the mechanical stresses caused by heart contractions without impairing its function. In addition, the biomaterial used must be biodegradable and have a degradation rate that corresponds to the rate of the new formation of the extracellular matrix (ECM) [5].

Despite significant advances, many biomaterials still exhibit lower conductivity, and their fibers are typically 400–600 nm in diameter, which differs from the size of native collagen fibers [6]. This discrepancy in properties can lead to suboptimal results in tissue regeneration as the cells cannot properly adhere or communicate electrically with the scaffold.

One of the most commonly used methods to produce membranes for biomedical applications is electrospinning [7]. This technique is valued for its versatility, as it allows researchers to control various parameters such as the fiber diameter, porosity and material composition. Electrospinning enables the production of a range of polymers, depending on the type of solvent used and the polymer concentration [8]. This adaptability makes it an ideal method for the production of scaffolds that mimic the native ECM of cardiac muscle [6].

One of the most promising biomaterials is collagen type I, a natural protein that is abundant in human tissue [3]. In cardiac muscle, collagen is an essential component of the ECM, where it forms fibers with diameters between 100 and 300 nm [9]. However, collagen itself lacks conductivity, which limits its application in electrically active systems, such as the heart or the nervous system. To overcome this limitation, collagen can be modified by incorporating ions between its polymer chains, which improves its ability to conduct electrical signals [10].

In the synthesis of collagen (C) nanofibers by electrospinning, solvents such as hexafluoroisopropanol (HFP) or trifluoroacetic acid (TFA) are usually used [11]. These solvents are effective in dissolving collagen due to their ability to form hydrogen bonds with proteins [12,13]. However, due to the strong interaction between these solvents and the collagen chains, it is difficult to completely remove the solvent residues, which may affect the chemical properties and biocompatibility of the final membrane. Nevertheless, the potential toxicity and environmental concerns associated with HFP and TFA have prompted researchers to investigate alternative solvents [14].

For example, phosphate-buffered saline (PBS) and ethanol have been shown to be effective in the electrospinning of collagen and offer a safer and more environmentally friendly approach [15]. These solvents are non-toxic and biocompatible and allow the production of collagen nanofibers with structural properties comparable to those obtained with more harmful solvents [13].

The incorporation of conductive materials into collagen membranes offers an interesting opportunity to improve the ionic and electrical conductivity of the material, making it suitable for neural and cardiac tissue engineering applications [16]. Electrical conductivity can be achieved by incorporating metallic nanoparticles or carbon-based materials [17], but conductive polymers are particularly attractive candidates [18]. Conductive polymers not only provide electrical conductivity but also enable the transport of ions, which is crucial for mimicking the natural behavior of excitable tissues such as the heart and nerves [19].

Two of the most intensively researched conductive polymers are polyaniline (Pani) and polypyrrole (Ppy) [20]. Both are considered biocompatible and biodegradable, which makes them suitable for use in bioelectronic devices. However, it is important to pay attention to their synthesis to avoid any cytotoxicity [21]. For example, the protonation of the NH_2_ groups in polyaniline is essential for maintaining conductive properties [22]. Reducing protonation can significantly minimize cytotoxicity, making it more suitable for biological applications [23]. An effective approach to achieve this balance is the chemical synthesis of conductive polymers using surfactants such as cetyltrimethylammonium bromide (CTAB) or sodium dioctylsulfoccinate (SDS) [24]. These surfactants help to regulate the protonation process and ensure that the polymer retains its emeraldine structure, the most conductive form of Pani, while improving its biocompatibility [10].

The aim of this study is to prepare, physicochemically characterize and test the biocompatibility of membranes made of type I collagen and conductive polymers using environmentally friendly synthesis methods, such as non-toxic solvents and surfactants, and to evaluate their potential for ion conduction in biomedical applications, especially in cardiac tissue regeneration.

## 2. Materials and Methods

### 2.1. Materials

Collagen (C) was extracted from bovine tendons according to previous publication [25]. Medium-molecular-weight chitosan (Ch) (190,000–310,000 Da, with 75% deacetylation), acetic acid, PBS, ethanol, calcium chloride (CaCl_2_), sodium chloride (NaCl) and tris(hidroximetil)aminometano (Tris) were from Sigma-Aldrich (Saint Louis, MO, USA). Polyaniline (Pani) and polypyrrole (Ppy) were polymerized with aniline, pyrrole ammonium persulfate, ferric (III) chloride (FeCl_3_), cetyltrimethylammonium bromide (CTAB), dioctyl sulfoccinate sodium (DSC) and hydrochloric acid from Sigma-Aldrich (Saint Louis, MO, USA).

### 2.2. Polymerization

The polymerization of Pani was carried out in a solution containing 0.055 M FeCl_3_ and 0.5 M HCl. Monomeric aniline was added together with CTAB and SDS, and the mixture was stirred until homogenization was achieved. Ammonium persulfate was then added dropwise to start the polymerization process. For PPy, the polymerization consists of preparing a homogeneous solution of pyrrole, CTAB and SDS, which is mixed ultrasonically. FeCl_3_ was then added dropwise to the mixture. Both solutions were left for 24 h under magnetic stirring. After this time, the resulting products were thoroughly washed with water and ethanol to remove impurities and surfactant residues [26,27].

### 2.3. Electrospinning

To prepare the polymer solutions, C was dissolved at a concentration of 340 mg/mL in a mixture of PBS and ethanol in a 1:1 ratio [13]. In parallel, a 0.4% Ch solution was prepared in acetic acid. Then C and Ch were mixed in a ratio of 4:1 with magnetic stirring overnight at room temperature. Pani and Ppy were dissolved separately in dimethylformamide (DMF) as a solvent. Once the solutions of each component were obtained, they were mixed until they formed a homogeneous solution [28,29].

A Fluidnatek Le-50 model (Nanofaber Srl, Rome, Italy) was used for electrospinning, with the intensity set to a variable voltage between 15 and 21 kV. The flow rate of the solution was set to 0.1 to 0.5 mL/h, and the distance between the static collector and the needle was set to 8 to 12 cm. The experiments were carried out at a humidity of less than 60% and at room temperature.

### 2.4. Characterization

#### 2.4.1. Morphology

The nanofibers were subjected to scanning electron microscopy (SEM) to observe the surface morphology and diameter of the fibers. A Helio 5 UC (Thermo Scientific, Monza, Italy) and a JSM-7401F (JEOL, Mexico City, Mexico) were used at an accelerating voltage of 15 kV and a magnification of approximately x3500, x5000 and x6500.

#### 2.4.2. Fourier Transform Infrared Spectroscopy (FTIR)

Fourier transform infrared spectroscopy (FTIR), a Nicolet iS50 FT-IR (Thermo Scientific, Mexico City, Mexico), was used to determine the chemical composition and integration of the polymers.

#### 2.4.3. BET Analysis of Porosity

An ASAP 2020 surface area and porosity analyzer (Micromeritics, Norcross, GA, USA) was used and the Brunauer–Emmett–Teller (BET) method was applied to estimate surface area and pore volume.

#### 2.4.4. Mechanical Characterization

To determine the modulus of elasticity of the membranes, a DMA 1 Start system (Mettler Toledo, Milan, Italy) was used for stress–strain analysis, with an increase of 0.1 N/min from 0.1 N to 5 N at 37 °C. The membranes were cut into probes of approximately 5 mm × 10 mm and the thickness of each sample was measured.

#### 2.4.5. Electrical Conductivity

Two different methods were used: A four-point probe was used to measure the electrical conductivity of the samples using a Keithley Model 2450 (SourceMeter, Mexico City, Mexico). The resistivity of the samples was determined by passing a constant current through the other probes and measuring the voltage through the inner probes. The volume resistivity was calculated as follows

ρ = 4.25 (V/I),(1)

The electrical conductivity (σ) of each sample was calculated as the reciprocal of the resistivity (ρ). Three films were taken from each sample to perform the analysis [16,30].

#### 2.4.6. Ionic Conductivity

A type H cell was used to study the transport of Na^+^ and Ca^2+^ ions across the membranes. A 50 mM Tris buffer solution was used on both sides of the cell, adjusted to a pH of 7.4 and maintained at 37 °C to simulate physiological conditions. Solutions of NaCl or CaCl_2_ were added to the left chamber of the cell to analyze the passage of ions across the membrane. Linear scanning voltammetry (LSV) was performed from −100 to 100 mV with two Pt electrodes in an SP-300 potentiostat (Biologic, Mexico City, Mexico). Impedance spectroscopic (EIS) measurements were performed with a 10 mV signal over a frequency range from 1 Hz to 1 MHz to evaluate the ionic conductivity and performance of the membrane [31]. The ionic conductivity σ was determined using the following equation:

σ = l/(R∙A),(2)

#### 2.4.7. Cell Viability Evaluation

Dulbecco’s Modified Eagle Medium (DMEM) and fetal bovine serum (FBS) were from Sigma-Aldrich (Saint Louis, MO, USA); Alamar Blue and Phalloidin Alexa Fluor 540 were from Invitrogen (Waltham, MA, USA).

The membranes were placed in a 96-well plate covering the entire surface and sterilized by washing three times with 70% ethanol for 5 min each time and UV light for 15 min per side [9]. H9c2 cells were seeded at a density of 3000 cells/mm^2^ on the membranes in DMEM media containing 10% FBS and incubated for 1 and 3 days at 37 °C and 5% CO_2_. At the designated time points, Alamar Blue was added at a concentration of 10% and incubated for 2 h. The media were collected and centrifuged, and the supernatant was measured by fluorescence measurement at 530/590 nm on a BioTek Synergy HT microplate (Bio-Tek, Winooski, VT, USA) [32]. Experiments were performed in triplicate (n = 3). For statistical analysis, a two-way ANOVA was performed, with statistical significance set at *p* < 0.05. For preliminary assessment of cell adherence, H9c2 cells were fixed with 4% paraphormaldehyde and phalloidin. Alexa Fluor 540 was used to stain the cytoskeleton on the membrane surface [33]. This was achieved using a Leica TCS SP5 confocal microscope with a D-Apochromatic 40X, 1.2 NA and oil objective (Leica Microsystems, Mexico City, Mexico) at 25 °C.

## 3. Results

### 3.1. Fourier Transform Infrared Spectroscopy

**Figure 1:**
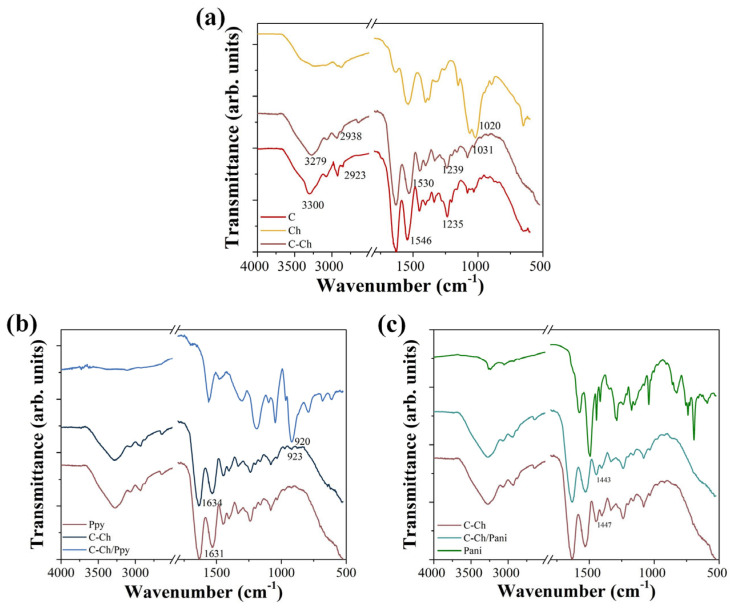
(**a**) FTIR spectra showing intermolecular interactions between collagen (C) and chitosan (Ch) due to hydrogen bonding via amine and hydroxyl functional groups. (**b**) FTIR spectra of C-Ch membranes with Ppy indicating a shift in the secondary amine region. (**c**) C-Ch membranes in combination with Pani show no significant spectral shifts, suggesting minimal interactions.

The samples were analyzed using Fourier transform infrared spectroscopy (FTIR) and the resulting spectra are shown in Figure 1. The characteristic functional groups of the type I collagen can be seen in Figure 1a, where the signals of the amide I group (1631 cm^−1^), amide II (1530 cm^−1^) and amide III (1239 cm^−1^) stand out. These signals provide information about the tertiary structure of the protein, as previously reported [34,35].

The addition of chitosan led to shifts in the absorption bands at 3279, 1530 and 1239 cm^−1^, which are attributed to the OH and NH_2_ groups that can interact with the NH_2_ collagen groups located outside the triple helix and form hydrogen bonds [36,37]. Moreover, the observed shifts at 1031 cm^−1^, 1530 cm^−1^ and 3279 cm^−1^ correspond to the presence of chitosan interacting with the triple helix structure of the collagen. This interaction leads to stresses in the glycosidic group, suggesting a hydrogen bonding interaction between the NH group of anime II and the OH groups [38].

The spectra of the individual components and the resulting composite with Ppy are shown in Figure 1b. The characteristic signals of Ppy include absorption bands at 1689 cm^−1^, corresponding to CH_2_, and at 1559 cm^−1^ and 1480 cm^−1^, which are associated with the pyrrole ring stretching modes of C=C and C–N, respectively [39]. Additional signals at 1304 cm^−1^ and 1046 cm^−1^ correspond to =C–H bending, while 1188 cm^−1^ is related to the C–H reduction in the polymer. A characteristic C=C oscillation is also observed at 920 cm^−1^ [40,41].

When Ppy is mixed with collagen, the characteristic signals of collagen are preserved, indicating that the tertiary structure of the protein remains intact (Figure 1c). However, an increased intensity and a shift at 923 cm^−1^ are observed, which is due to the incorporation of Ppy into the mixture. In addition, a left shift is observed at 1634 cm^−1^, which is probably due to the strain of the primary amide group as a result of the interaction with the protonated N–H group of the pyrrole ring [29].

In the case of Pani, shown in Figure 1c, a signal at 1579 cm^−1^ is attributed to the quinoid ring with C=C stretching, while the signal at 1492 cm^−1^ is associated with the benzoid ring [42]. The presence of both signals confirms the formation of the emeraldine salt of Pani [43]. Additional signals at 1287 cm^−1^ are characteristic of the secondary ring structure attributed to the C–N stretching, along with bands at 1172 cm^−1^ and 826 cm^−1^ related to the π-polaron resonance [44,45]. In addition, C–H signals are observed at 1445 cm^−1^, 1415 cm^−1^ and 739 cm^−1^ [46].

When Pani was mixed with collagen, the characteristic triple helix structure of the collagen was preserved. However, a shift to the right at 1443 cm^−1^ and changes in the relative intensity of the signal at 1400 cm^−1^ were observed, which corresponds to C–H stretching [47]. These shifts indicate a weak interaction between Pani and collagen, probably due to the minimal direct binding or interference between the polymer and the protein.

### 3.2. Scanning Electron Microscopy

**Figure 2:**
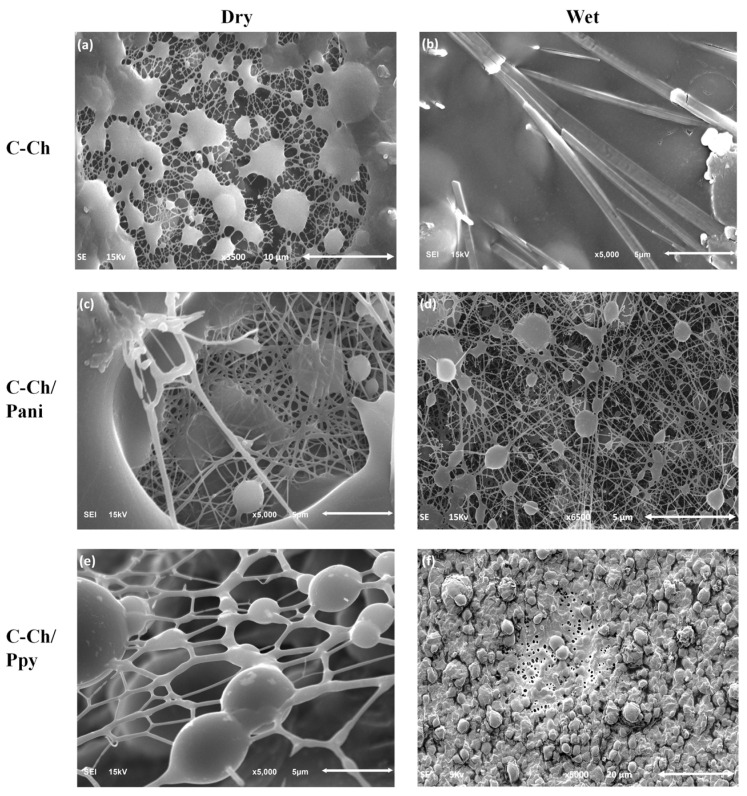
SEM images of the dried membranes of (**a**) C-Ch, (**c**) C-Ch/Pani and (**e**) C-Ch/Ppy as well as the membranes after wetting of (**b**) C-Ch, (**d**) C-Ch/Pani and (**f**) C-Ch/Ppy, showing the loss of morphology due to swelling.

The membranes produced using the electrospinning technique can be seen in Figure 2, which shows the dried membranes after electrospinning. The dimensions of the fibers were analyzed using Image J 1.54, with statistics of 100 measurements for each sample. In Figure 2a, the C-Ch membrane has a fiber diameter of 105 ± 34 nm, accompanied by bean structures around the fibers. The observed impurities, measuring 148 ± 16 nm, could be responsible for the significant loss of the fiber morphology after wetting (Figure 2b). Once Pani is added to the solution, the size of the fibers decreases to 71 ± 24 nm (Figure 2c). After wetting, the presence of small beans with a diameter of 298 ± 144 nm increases, and a deformation of the fibers can be observed in Figure 2d. The addition of Ppy leads to an increase in the fiber diameter to 394 ± 365 nm (Figure 2e) with a bean size of 3.3 µm, resulting in a complete loss of morphology after membrane wetting.

The electrospinning conditions for each solution correspond to a voltage of 19 kV, a solution flow of 0.1 mL/h and a distance between the collector and the needle of 12 cm. The observed loss of morphology in the samples can be attributed to swelling leading to the binding of the fibers and the formation of irregular aggregates or beads, as shown in Figure 2b,f. This phenomenon is likely the result of interactions between the functional groups within the materials, as shown in Figure 1b, suggesting the formation of hydrogen bonds and other intermolecular interactions that can disrupt the structural integrity of the fibers [48].

In the case of Figure 2d, which corresponds to the sample with Pani, the observed morphological changes could be due to the weak interaction between Pani and the C-Ch network. The absence of a strong bond may lead to local swelling or the collapse of the fiber network, causing the fibers to fuse together and form beads.

This morphological change underlines the sensitivity of the composite structure to variations in environmental conditions or polymer interactions and highlights the need to optimize the balance between the swelling resistance and mechanical stability of the membranes for specific applications.

### 3.3. Porosity

The membranes were evaluated by the absorbance of nitrogen to obtain the porosity per volume of the membranes as a control of the porosity of membranes of collagen–chitosan from a previous work [25], with a pore size of 32.40 nm and a surface area of 4.49 m^2^/g.

C-Ch/Ppy showed a BET surface area of 31.47 ± 30 m^2^/g, a pore size of 1.079 ± 0.09 nm and a particle size of 2.00 ± 1.9 µm. The C-Ch/Pani membrane has a BET surface area of 0.0097 ± 0.008 m^2^/g and an average particle size of 325.5 ± 20.4 µm.

### 3.4. Mechanical Properties

The mechanical properties of the membranes were investigated at 37 °C to simulate physiological conditions, with a thickness of approximately 50 µm [49], as shown in Table 1. The Young’s moduli of the membranes are consistent with the values required in a myocardial tissue between 0.2 and 0.5 MPa [50]. As can be seen in the graph, the addition of Pani increases the ε value by 50, which is due to the presence of beans between the fibers as well as their dispersion, which allows for a better distribution of forces. At the same time, the presence of Ppy leads to continuous beans in the fibers, which reduces the modulus of elasticity.

**Figure 3:**
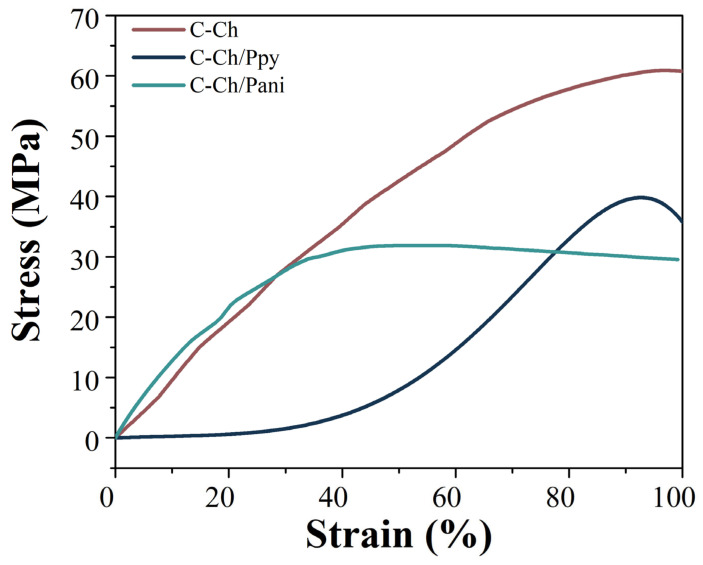
The stress–strain curve of the membranes of the precursors (C-Ch) and C-Ch/Pani, which show a typical plastic behavior; C-Ch/Ppy, on the other hand, shows an elastomeric curve.

The behavior of C-Ch and C-Ch/Pani in the elastic range is comparable, as illustrated in Figure 3. However, the incorporation of Pani reduces the tensile strength [51]. This phenomenon can be attributed to a subtle interaction between the polymers that prevents the collagen network from resisting the load in the same way, although its elasticity improves at lower load values. These weak interactions can disrupt the inherent organization of collagen fibers and reduce their ability to distribute loads evenly. Despite the decrease in the tensile strength, the composite exhibits an improved elasticity at lower stress levels, suggesting that Pani may impart flexibility to the material by partially affecting the stiffness of the collagen structure. Compared to some previously reported values, these membranes have lower ε-values. However, it is important to note that they were also significantly thicker than the fibers described in these studies [9,47].

On the other hand, the addition of Ppy changes the mechanical performance considerably, as can be seen in Figure 3, where it behaves more like an elastomer. The ε values obtained for these membranes fall within the range previously reported for similar biomaterials [48,52].

This elastomeric change can be attributed to the electrostatic interactions between the polymers, especially with their N–H groups, which allow the network to distribute stresses more effectively.

These interactions allow for a better stress distribution across the network and improve its ability to resist deformation while maintaining structural integrity. The shift observed in the FTIR spectra (Figure 2b, 923 cm^−1^ and 1634 cm^−1^) indicates the presence of intermolecular forces between the Ppy and collagen chains. The elastomeric behavior would allow the material to withstand the continuous contraction and relaxation cycles of the heart without mechanical failure.

### 3.5. Conductivity

The electrical conductivity values can be found in Table 2. It can be observed that the addition of Pani leads to a significant increase in conductivity. This performance can be attributed to the fact that collagen does not affect the conductivity of Pani, so it does not act as an electrical insulator. The signals in Figure 2c show that the conductive emeraldine salt structure of Pani is maintained even in the presence of collagen and enables efficient charge transport.

In contrast, the C-Ch/Ppy composite shows a significant decrease in electrical conductivity. This decrease can be attributed to the interactions between Ppy and Col layers, which can interfere with the electron transport mechanisms of Ppy. In particular, the NH groups of Ppy, which play a crucial role in the delocalization and transport of electrons, can be partially blocked due to hydrogen bonding or electrostatic interactions with the collagen matrix [48]. The structural change in the Ppy matrix, probably caused by its incorporation into the collagen network, impairs the conductive properties.

Compared to other membranes, the conductivity was higher than [53,54] and lower than [28,50] and was between the required conductivity in this tissue.

**Figure 4:**
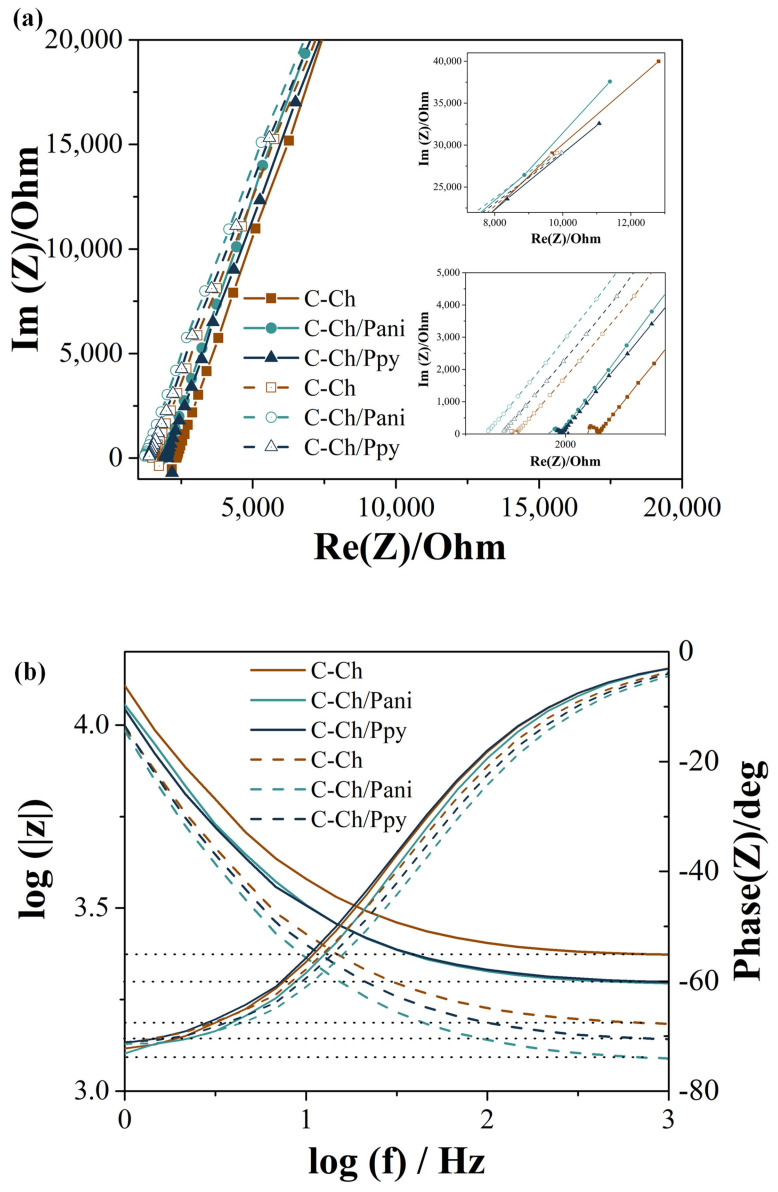
(**a**) Impedance using calcium (fill symbols) and sodium (symbols with border) and (**b**) Bode impedance using calcium (solid lines) and sodium (dashed lines).

In terms of ionic conductivity, Figure 4 shows the EIS analysis showing a diffusion-limited process and a comparison of the Nyquist plots of the membranes to explain the behavior of the charge transfer resistance (Rct) [55]. The Rct for the Na^+^ transfer of C-Ch is 8324 Ω, C-Ch/Pani is 8431 Ω and C-Ch/Ppy is 8582 Ω. In contrast, the Rct for Ca^2+^ is 10,573 Ω for C-Ch, 9441 Ω for C-Ch/Pani and 9120 Ω for C-Ch/Pani.

The results indicate that the calcium transfer has higher Rct values compared to sodium, which could be due to the divalent charge of Ca^2+^ and the larger ionic radius that reduces its mobility due to interactions with negatively charged functional groups in the C-Ch matrix. The resistance trends observed in the EIS analysis were consistent with the LSV results shown in Table 2. This confirms that the membranes regulate the ion transport, especially for calcium ions, in a similar way to the extracellular matrix and allow a controlled ion distribution [56].

The Bode diagram in Figure 4b analyzes the impedance value (∣Z∣) and phase angle (θ) as a function of the logarithmic frequency and provides insight into the electrochemical behavior of the membranes. The decrease in ∣Z∣ with the increasing frequency is characteristic of resistive and capacitive systems [51]. At low frequencies (log f < 1), the high impedance indicates that Rct dominates, suggesting that ionic transport is limited by faradaic processes and the electrode–electrolyte interface. As the frequency increases (log f > 2), the impedance drops sharply and enters a range that is dominated by the solution resistance and the double-layer capacitance [57].

The variation in the phase angle additionally supports this performance. At low frequencies, values close to −80° indicate a capacitive response where ions accumulate at the interface without efficient conduction taking place. With the increasing frequency, a transition around log f ≈ 1.5 marks the transition from charge-transfer-dominated processes to diffusion-controlled transport. At higher frequencies (log f > 2.5), the phase angle stabilizes near 0°, indicating resistive behavior dominated by the electrolyte resistance [58,59].

The incorporation of Pani and Ppy into the C-Ch matrix reduces the overall impedance and increases the ionic conductivity. However, these polymers also change the charge distribution and ion interactions, especially at intermediate frequencies. The lower impedance at low frequencies in membranes doped with Pani and Ppy indicates an improved charge transfer and lower energy barriers for the ion transport [60,61]

Overall, the results confirm that the Ca^2+^ transport encounters a higher resistance than the Na^+^ transport, which is reflected in the increased impedance at low frequencies. Conducting polymers modulate the electrochemical response of membranes, improve ionic conductivity and regulate the ion–matrix interactions.

### 3.6. Cell Viability

**Figure 5:**
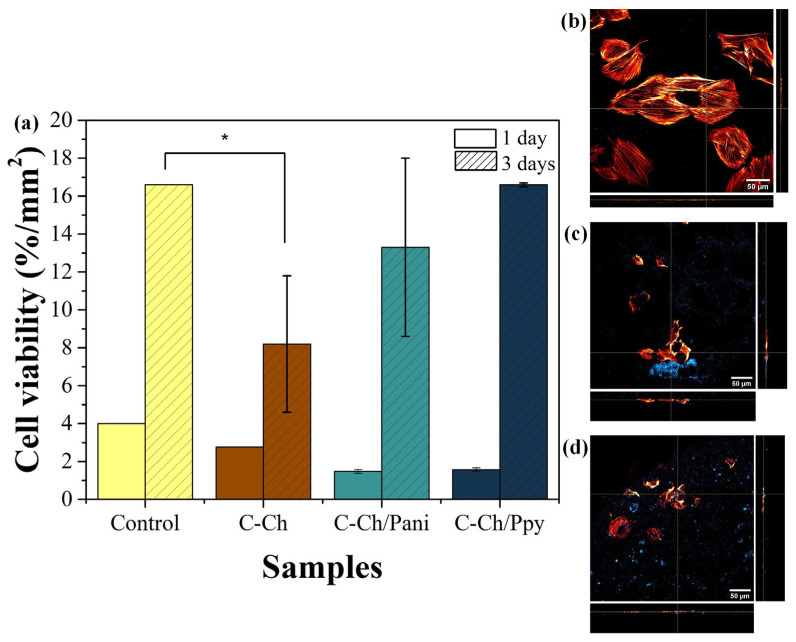
(**a**) The evaluation of the cell viability of membranes assessed after 1 and 3 days in contact with myoblasts H9c2 (n = 3) using a two-way ANOVA where * indicates statistical significance difference, and the standard error was plotted. (**b**) The fluorescence of the cytoskeleton after 1 day of the control sample. In the cells with (**c**) C-Ch/Pani and (**d**) C-Ch/Ppy; the blue fluorescence is due to the membrane.

Figure 5 shows the cell viability of the membranes in contact with the myoblast H9c2. Figure 5a shows significant differences in the C-Ch membrane on the third day, in contrast to C-Ch/Pani and C-Ch/Ppy, whose viability does not differ from the control sample. These results indicate that the membranes obtained do not exhibit cytotoxicity [62].

The preliminary assessment of the adhesion of myocytes to the membrane surface after one day of contact with H9c2 in C-Ch/Pani and C-Ch/Ppy is shown in Figure 5c and Figure 5d, respectively. Both images show the adhesion of the cells to the membrane surface, with the observed blue fluorescence being due to the intrinsic fluorescence of the membrane. However, the cytoskeletons appear smaller than in the control sample (Figure 5b), indicating an initial stress response. This observation is confirmed by Figure 5a after 1 day, which is probably due to the adaptation phase of the cells to the biomaterial surface [63].

## 4. Discussion

The integration of conductive polymers, such as Pani and Ppy, into C-Ch matrices has shown significant effects on the structural, mechanical and electrical properties of the resulting composites. The Pani samples exhibited an increased electrical conductivity due to the minimal interaction between Pani and the C-Ch network, which preserves the conductive pathways of Pani. However, this weak interaction also led to a decrease in tensile strength as the polymer disrupted the inherent structural resistance of the collagen network. Nonetheless, the composite showed an improved elasticity at lower stress values, making it suitable for applications where flexibility takes precedence over mechanical strength.

In contrast, the addition of Ppy led to stronger electrostatic interactions with the collagen matrix, which improved the stress distribution and gave the material a more elastic behavior, but at the cost of lower electrical conductivity due to a partial obstruction of the electron transport mechanisms of Ppy. Morphologically, swelling effects in both composites led to fiber bonding and bead formation, but these were more pronounced in the Ppy samples due to stronger interactions within the polymer network.

These results indicate a greater resistance to diffusion from Ca^2+^ than Na^+^, a result expected due to the larger ionic radius and divalent charge of Ca^2+^, which promotes stronger interactions with the negatively charged functional groups of the C-Ch membrane. Similarly, the interactions in C-Ch/Ppy allow for a lower ionic resistance compared to C-Ch/Pani, where the functional groups remain more accessible and prevent ion mobility.

A preliminary assessment of the viability and adherence of H9c2 cells underpinned the influence of the polymer choice on the membrane behavior. However, the smaller cytoskeletons observed compared to the control sample suggest that the cells take longer to stabilize under conductive polymers or that a differential volumetric adaptation occurs due to the inherent roughness of the polymers compared to the relatively flat surface of a well plate. This was particularly evident in C-Ch/Ppy, where stronger electrostatic interactions may have influenced the organization of the cytoskeleton in the early stages.

These results underline the potential of C-Ch/Pani for electrically active scaffolds and of C-Ch/Ppy for dynamic mechanical applications. At the same time, it becomes clear that further optimization is required to achieve a balance between conductivity, mechanical integrity and morphological stability for the desired viability and adherence of cells to the surface of membranes.

## 5. Conclusions

The integration of conductive polymers into C-Ch matrices led to significant differences in electrical, mechanical and structural properties, depending on the polymer incorporated. The incorporation of Pani resulted in a composite with an electrical conductivity of 5.18 × 10^−4^ S/cm, mainly due to its limited interaction with the collagen network, which retains its conductive pathways. It also exhibits a Na ion conductivity of 5.47 × 10^−9^ S/cm, possibly influenced by a low BET surface area of 0.0097 ± 0.008 m^2^/g. However, this was at the expense of tensile strength, although the material exhibited a 47.5% higher elasticity at low stresses. In addition, the composite showed a promising non-cytotoxic performance after 3 days.

In contrast, Ppy formed stronger electrostatic interactions with the C-Ch matrix, resulting in more elastic behavior and improved stress distribution, but with an 80% decrease in electrical conductivity to 1.79 × 10^−4^ S/cm, likely due to partial impairment of electron transport mechanisms. Conversely, the Na ion conductivity was threefold higher at 6.23 × 10^−9^ S/cm, possibly related to the significantly large BET surface area of 31.47 ± 30 m^2^/g. The C-Ch/Ppy composites exhibited a cell viability of 14%/mm^2^ after 3 days, emphasizing their potential as scaffold material.

Morphological changes, including swelling and bead formation, were observed in both systems, highlighting the need for further refinement to ensure structural stability. These results highlight the potential of C-Ch/Pani composites for electrically responsive frameworks and of C-Ch/Ppy composites for applications requiring mechanical flexibility. However, future efforts should focus on optimizing the interplay between electrical performance, mechanical integrity and biocompatibility to develop tailored biomaterials for specific tissue regeneration applications.

## Figures and Tables

**Table 1:** Young’s module of membranes evaluated at 37 °C.

Membrane	Membrane Thickness (µm)	Young’s Modulus, ε (Mpa)
C-Ch	47	0.882
C-Ch/Pani C-Ch/Ppy	82 51	1.18 0.025

**Table 2:** Conductivity of membranes of C-Ch with Pani and Ppy.

Membrane	Electrical Conductivity (S/cm)	Ionic Conductivity, Na^1+, 1^ (S/cm)	Ionic Conductivity, Ca^2+, 2^ (S/cm)
C-Ch	2.69 × 10^−4^	1.68 × 10^−10^	6.64 × 10^−9^
C-Ch/Pani C-Ch/Ppy	5.18 × 10^−4^ 1.79 × 10^−4^	5.47 × 10^−9^ 6.23 × 10^−9^	4.2 × 10^−9^ 6.56 × 10^−9^

^1^ NaCl solution was used; ^2^ CaCl_2_ solution was used.

## Data Availability

The original contributions presented in this study are included in the article. Further inquiries can be directed to the corresponding authors.
